# Osteoprotegerin Gene Polymorphisms Are Associated with Subclinical Atherosclerosis in the Mexican Mestizo Population

**DOI:** 10.3390/diagnostics12061433

**Published:** 2022-06-10

**Authors:** Benny Giovanni Cazarín-Santos, Nonanzit Pérez-Hernández, Rosalinda Posadas-Sánchez, Gilberto Vargas-Alarcón, Óscar Pérez-Méndez, Juan Rodríguez-Silverio, Bladimir Roque-Ramírez, Verónica Marusa Borgonio-Cuadra, José Manuel Rodríguez-Pérez

**Affiliations:** 1 Departamento de Biología Molecular, Instituto Nacional de Cardiología Ignacio Chávez, Mexico City 14080, Mexico; giovanni.cazarin@gmail.com (B.G.C.-S.); gvargas63@yahoo.com (G.V.-A.); opmendez@yahoo.com or; 2 Sección de Estudios de Posgrado e Investigación, Escuela Superior de Medicina, Instituto Politécnico Nacional, Mexico City 11340, Mexico; jrsilverio61@yahoo.com.mx; 3 Departamento de Endocrinología, Instituto Nacional de Cardiología Ignacio Chávez, Mexico City 14080, Mexico; rossy_posadas_s@yahoo.it; 4 Tecnologico de Monterrey, Campus Ciudad de Mexico, Mexico City 14380, Mexico; 5 Laboratorio de Nutrigenética y Nutrigenómica, Instituto Nacional de Medicina Genómica (INMEGEN), Mexico City 14610, Mexico; broque@inmegen.gob.mx; 6 Departamento de Medicina Genómica, Instituto Nacional de Rehabilitación Luis Guillermo Ibarra Ibarra, Mexico City 14389, Mexico; vborgoni@yahoo.com.mx

**Keywords:** osteoprotegerin, subclinical atherosclerosis, genetic susceptibility, single nucleotide polymorphism, calcification

## Abstract

Subclinical atherosclerosis (SA) is the presence of coronary calcification in the absence of cardiovascular symptoms, and it usually progresses to atherosclerotic disease. Studies have shown an association of osteoprotegerin gene (*OPG*) variants with calcification process in cardiovascular diseases; however, to this day there are no studies that evaluate individuals in the asymptomatic stage of atherosclerotic disease. Therefore, the purpose of this study was to analyze the association of four genetic variants and haplotypes of the *OPG* gene with the development of SA, through TaqMan genotyping assays. We also aimed to identify potential response elements for transcription factors in these genetic variants. The study included 1413 asymptomatic participants (1041 were controls and 372 were individuals with SA). The rs3102735 polymorphism appeared as a protective marker (OR = 0.693; 95% CI = 0.493–0.974; *p*_heterozygote_ = 0.035; OR = 0.699; 95% CI = 0.496–0.985; *p*_codominant_ _1_ = 0.040) and two haplotypes were associated with SA, one as a decreased risk: *GACC* (OR = 0.641, 95% CI = 0.414–0.990, *p* = 0.045) and another as an increased risk: *GACT* (OR = 1.208, 95% CI = 1.020–1.431, *p* = 0.029). Our data suggest a lower risk of SA in rs3102735 *C* carriers in a representative sample of Mexican mestizo population.

## 1. Introduction

Subclinical atherosclerosis (SA) is defined as the presence of coronary calcification, in the absence of cardiovascular symptoms [1]. This process develops silently over the years and usually advances to a high degree when clinical symptoms appear, such as an acute coronary event. Currently, coronary artery calcification (CAC) is considered an early marker of coronary artery disease (CAD) [1,2] and the use of CAC score is one of the main approaches for a primary detection of SA in apparently healthy individuals [3,4]. Moreover, it has been reported that the calcification of coronary arteries represents a risk factor for adverse cardiovascular events [5].

The development of atherosclerotic plaques and the presence of coronary calcification is determined by a complex and still unclear interaction between traditional risk factors and genetic components [6]. The genetic approach in cardiovascular diseases has revealed polymorphisms with functional repercussions on the encoded proteins that have been associated with vascular diseases. Among these proteins, osteoprotegerin (OPG) is considered a main contributor of the vascular calcification processes [7]. The human *OPG* gene is located at locus 8q24.12, it contains five exons [8] and encodes for a 401-amino acid glycoprotein with a molecular weight of 60 kDa. OPG belongs to the tumor necrosis factor superfamily and contains seven domains implicated in its biological activities [7,9,10]. OPG is produced in vascular smooth muscle cells and vascular endothelial cells, among others [9,10]; it has been proposed as regulator of vascular diseases, vascular calcification and bone matrix homeostasis [11,12].

Different studies have shown an increased expression of OPG in carotid atherosclerotic lesions [13], as well as in epicardial adipose tissue of patients with coronary artery disease [14]. Moreover, elevated levels of OPG in serum have been associated with subclinical and vascular diseases [10,15,16,17,18]. To-date, few studies have investigated the genetic association of *OPG* gene and the presence of SA; furthermore, the results have been paradoxical [19,20]. Thus, the aim of the present study was to investigate whether the genetic variants rs2073618 (G1181C), rs3134069 (C209T), rs3134070 (T245G), rs3102735 (A163G) and haplotypes of *OPG* gene are associated with the risk of developing SA. Also, we performed an in silico analysis in order to identify the potential functional effects of these genetic variants.

## 2. Materials and Methods

### 2.1. Study Population

This research is a cross-sectional study performed during the baseline evaluation of individuals recruited for the Genetics of Atherosclerotic Disease study (Spanish acronym GEA). The GEA study was designed at the Instituto Nacional de Cardiología Ignacio Chávez (INCICh) to determine the genetic bases of CAD and to evaluate its association with conventional and emerging cardiovascular risk factors [21].

The study population included a total of 1413 unrelated, clinically healthy, asymptomatic participants, with no personal or family history of CAD. These participants were blood donors attending the blood bank of the INCICh or recruited by open invitations in different social services centers from 2008 to 2013, in Mexico City. The participants were characterized by biochemical determinations, medical history, demographics, anthropometry and nutritional information collected through standardized and validated questionnaires [21,22,23,24]. For this study, the exclusion criteria were: the presence of heart or renal failure, thyroid disorders, and liver and oncological diseases.

The participants underwent a chest and abdomen axial tomography using a 64-channel multi-detector helical computed tomography (CT) system (Somaton Sensation Siemens, Forcheim, Germany). After assessment of CT, CAC score was determined by the Agatston method [25]; a CAC score > 0 in the absence of coronary symptoms was defined as SA. Then, 372 individuals were identified with SA, whereas 1041 individuals with a CAC = 0 formed the control group.

The present study is in compliance with the Helsinki Declaration and was approved by the Ethics and Research Committees of the INCICh (Register number 18-1071). All enrolled participants provided a written informed consent.

### 2.2. Single Nucleotide Polymorphism (SNPs) Selection and Genotyping

Genomic DNA was extracted from peripheral blood using standard methods (DNA Blood Mini kit, QIAGEN, Hilden, Germany). All polymorphisms were genotyped using specific TaqMan assays on a Prism 7900HT Fast Real-Time PCR system following the supplier’s instructions (ThermoFisher Scientific, Foster City, CA, USA).

The polymorphic sites were selected by a prior review of the NCBI data base, and included polymorphisms with a minor allele frequency (MAF) >5%, previously demonstrated to be associated with cardiovascular diseases and with vascular calcification.

### 2.3. Bioinformatics Analysis of Prediction of Transcription Factor Bindings Sites

To identify potential response elements for transcription factors as a consequence of polymorphisms in the *OPG* gene promoter, the in silico analysis was performed with the bioinformatics software tools PROMO version 3.0.2, website (http://alggen.lsi.upc.es/cgi-bin/promo_v3/promo/promoinit.cgi?dirDB=TF_8.3 (accessed on 12 March 2022)). This analysis was performed on flanking sequences of 25 bases upstream and 25 bases downstream for the two alleles of each polymorphism, with a dissimilarity margin less than or equal to 15% [26]. Also, SNPinfo website (https://snpinfo.niehs.nih.gov/snpinfo/snpfunc.html (accessed on 12 March 2022)) was employed and the analysis of SNPrsID corresponding to each polymorphism included the allele frequency data from all populations reported in HapMap and dbSNP [27].

### 2.4. Statistical Analysis

According to the data distribution, the continuous variables were expressed as mean ± standard deviation (SD) or median (interquartile range); the categorical variables were described as percentages. Either Student’s *t*-test or a Mann–Whitney U test was performed for the two-group comparisons of continuous variables whereas for the categorical variables the Pearson’s chi-squared test was used. In both study groups, the allele and genotype frequencies were obtained by direct count and the Hardy−Weinberg equilibrium was tested in the four polymorphisms. A logistic regression analysis was applied to determine the association of polymorphisms with subclinical atherosclerosis, under different inheritance models, adjusted by cardiovascular risk confounding variables. The construction and analysis of haplotypes were performed with the Haploview software v4.1 (Broad Institute of Massachusetts [MIT], Cambridge, MA, USA). Statistical analyses were performed using SPSS v20.0 (SPSS Inc., Chicago, IL, USA). A statistically significant value was established as *p* < 0.05.

## 3. Results

### 3.1. Characteristics of the Studied Groups

Clinical and metabolic characteristics as well as cardiovascular risk factors of the studied population are shown in Table 1. Statistically significant differences in individuals with SA were observed, when compared to the control group in terms of age, sex (males), body mass index, waist circumference, LDL-cholesterol, triglyceride and glucose levels (*p* < 0.05). Concerning cardiovascular risk factors, individuals with SA had higher frequencies of subcutaneous abdominal fat, LDL-cholesterol ≥ 130 mg/dL, and non-HDL cholesterol >160 mg/dL when compared to the control group (*p* < 0.05).

### 3.2. Association of OPG Gene Polymorphisms with Subclinical Atherosclerosis

**Figure 1:**
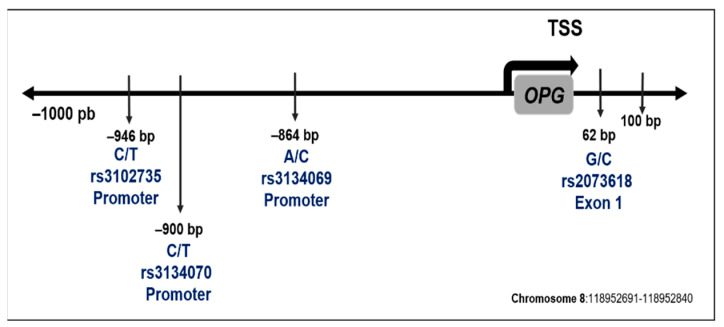
Location in the *OPG* gene sequence of selected polymorphisms. The position is with respect to the transcription start site (TSS).

Figure 1 describes the location of the evaluated polymorphisms. The sequence studied and MAF corresponding to each polymorphism are described in Table 2.

**Figure 2:**
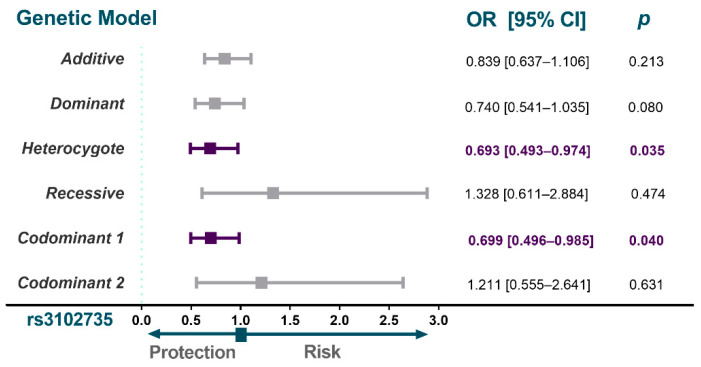
Association analysis of *OPG* gene polymorphisms with SA. The models shown were adjusted by sex, age, BMI, smoking habits, diabetes mellitus, LDL-cholesterol, subcutaneous abdominal fat, alkaline phosphatase, alanine transaminase and aspartate transaminase activities, apolipoprotein AI concentrations and phosphorus and calcium serum concentration. SA, subclinical atherosclerosis; MAF, minor allele frequency; OR, odds ratio; CI, confidence interval.

The expected and observed frequencies of the four polymorphisms in the whole sample of this study were in Hardy-Weinberg equilibrium. The genotype distributions are described in Figure 2. A different distribution of the *OPG* polymorphism rs3102735 was observed in individuals with SA when compared to the control group. Individuals with SA showed decreased frequencies of the *C* allele rs3102735 when compared to controls (OR = 0.693; 95% CI = 0.493–0.974; *p*_heterozygote_ = 0.035; OR = 0.699; 95% CI = 0.496–0.985; *p*_codominant 1_ = 0.040). Conversely, similar distributions of the *OPG* polymorphisms rs3134070, rs3134069 and rs2073618 were found in individuals with SA and controls. These analyses were adjusted by age, sex, body mass index, diabetes mellitus, subcutaneous abdominal fat, smoking habits, concentrations of apolipoprotein AI, LDL-cholesterol, calcium and phosphorus serum concentrations, as well as alkaline phosphatase, alanine transaminase and aspartate transaminase activities.

### 3.3. Haplotype Association Analysis of OPG Gene Polymorphisms

**Figure 3:**
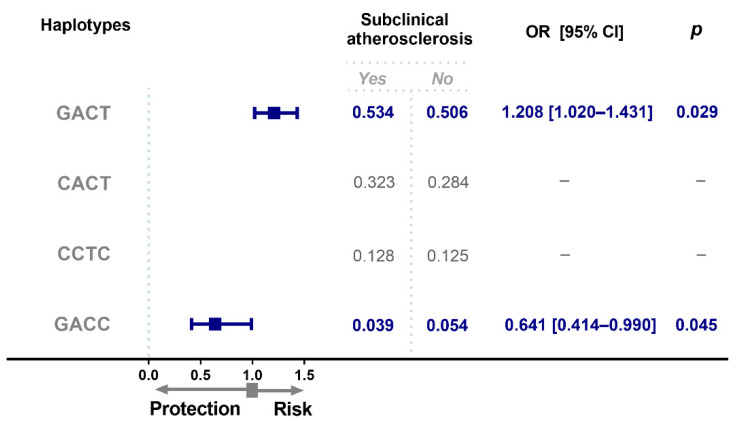
*OPG* haplotype frequencies in individuals with subclinical atherosclerosis. Haplotype analysis was performed based on the order and position of the polymorphisms in the chromosome. (rs2073618, rs3134069, rs3134070, rs3102735). OR, odds ratio; CI, confidence interval.

Two haplotypes (*GACT* and *GACC*) had different frequencies between groups (Figure 3); individuals with SA showed an increased frequency of the *GACT* haplotype when compared to the control group (OR = 1.208, 95% CI = 1.020–1.431, *p* = 0.029). While the *GACC* haplotype showed a decreased frequency in individuals with SA when compared to the control group (OR = 0.641, 95% CI = 0.414–0.990, *p* = 0.045).

### 3.4. In Silico Analysis of Transcription Factor Binding Sites to OPG Polymorphisms

The in silico analysis of response elements of transcription factors showed that rs3102735 modifies a DNA binding site; the substitution of cytosine for thymine causes the loss of the specific binding site for the enhancer-binding protein β (C/EBP-β) and the enhancer-binding protein α (C/EBP-α) factors.

### 3.5. Analysis of CAC Score between C Allele Carriers and Non-C Carriers

To explore whether the *C* allele of the rs3102735 polymorphism had any impact on coronary calcification, we compared the CAC score of patients with SA, grouped as *C* carriers and non-*C* carriers; this analysis showed a higher CAC score in non-*C* carriers than in *C* carriers, but this difference did not reach statistical significance (CAC = 34.30 [6.35–86.85], CAC = 24.60 [4.30–93.50], *p* > 0.05, respectively).

## 4. Discussion

In the present study, we assessed four polymorphisms of the *OPG* gene in Mexican individuals, in order to explore their possible association with SA. In only one polymorphism (rs3102735) a dissimilar distribution was observed in individuals with SA when compared to the control group. Currently, there are only a few studies involving the role of *OPG* gene polymorphisms with the presence of SA. Our data concerning the frequency of the rs3102735 polymorphism suggest a potential usefulness as a genetic marker of SA.

Nevertheless, the participation of rs3102735 as a susceptibility marker is still under debate. Even if this polymorphism has been shown to be associated with the incidence of diseases other than cardiovascular diseases [8,28,29], a lack of association with the incidence of symptomatic CAD [30] or large artery atherosclerosis stroke [28] has been reported. The topography of atherosclerotic lesions as well as the stage of the disease may be at the basis of these discrepancies.

Similarly, the analysis of *OPG* polymorphisms performed by Soufi et al. and Rhee et al., included rs3134069 and rs3102735 polymorphisms, and did not demonstrate any association with CAD in Caucasian and Korean populations, respectively [29,31]. Discrepancies concerning the association between *OPG* polymorphisms and cardiovascular diseases may be related to several circumstances, such as the number of patients included in every study and the technique used for genotyping and the number of SNPs evaluated. (a) We determined four SNPs and we used CT to evaluate the CAC score, whereas the report by Alkady et al. [32] was based in the intima–media thickness by echography as a subrogate of atherosclerosis and they only analyzed one polymorphism. Considering that OPG is involved in the calcification process in tissues, the lack of association in the study by Alkady et al. [32] is not surprising, whereas we defined SA by the presence of coronary calcification. (b) The report by Soufi et al. included just 468 male patients with and without CAD, as compared to the 1413 participants in our study. In addition, some genetic determinations were made by RFLP, whereas we used TaqMan genotyping assays in our study. (c) The study by Rhee et al. also included a small number of patients in comparison to our report. (d) There is a potential ethnic contribution to take into account, since all these studies were performed in populations whose genetic backgrounds differ from that of the Mexican mestizo population [33,34,35,36].

Furthermore, the known physiological role of OPG allows an acceptable interpretation of the contribution of the protein to SA [37]. During atherosclerosis progression, OPG expression decreases in blood vessels concomitantly with the increase in the receptor activator of NF-κB (RANK) ligand (RANKL) [38]. The interaction of RANKL and RANK activates the NF-κB pathway with the consequent transcription of pro-calcifying genes in VSMC, such as bone morphogenetic protein-4 [37]. OPG is a decoy receptor that binds to RANKL, thus preventing the interaction between RANK and RANKL [39], thus inhibiting the osteogenic differentiation of VSMC. Consequently, it is likely that OPG plays an anti-calcifying role that is consistent with increased levels of OPG during certain stages of the coronary heart disease, where OPG may be over-expressed to compensate for the deposition of calcium in the subendothelial space [14,37]. This idea is supported by the high serum levels of OPG in several vascular diseases and carotid intima–media thickness observed in patients with pathologies related to cardiovascular risk factors [40,41,42,43]. To gain more insight about the possible impact of the rs3102735 polymorphism, we performed an in silico analysis to establish whether there is a response element that could be affected by the substitution of *T* for *C* in the promoter sequence of the *OPG* gene. This approach suggests that the presence of the *C* allele, corresponding to MAF of the rs3102735, introduces a C/EBP response element that may enhance the expression of the *OPG* gene. C/EBPs are a group of transcription factors that belong to a superfamily formed by CREB, AP-1 and ATF that fulfill functions such as immune response and energy metabolism [44]. Therefore, the presence of the *C* allele of the rs3102735 may have a functional role in the expression of the *OPG* gene.

To further explore the potential impact of this polymorphism on coronary calcification, we compared patients with SA who were *C* carries to patients with SA who were non-*C* carriers. Even if the CAC score tended to higher values in non-*C* carriers, the difference did not reach statistical significance.

Furthermore, it is important to consider some limitations in our study: (a) our results provide a genetic approach to the potential contribution of osteoprotegerin but we did not establish whether the polymorphisms determine the protein expression; (b) age and gender are commonly associated with osteoprotegerin plasma levels, and our data cannot discard a potential interaction between *OPG* polymorphisms and these two characteristics; (c) additional studies are necessary to validate our findings with different ethnicities; (d) functional studies are required to clarify the role of *OPG* polymorphisms in the subclinical manifestation of atherosclerosis.

We recognize that our study was unpowered for subgroup comparisons; therefore, a large population study may be necessary to explore the potential influence of the *C* allele on CAC score. Moreover, the possibility that a neighboring gene co-segregates with the *OPG* gene, influencing the protective role described in this study, should not be discarded.

Concerning the haplotype analysis, the *GACT* haplotype was associated with an increased risk of having SA, whereas the *GACC* haplotype was associated with a decreased risk of presenting with subclinical disease. As expected, the main difference between the haplotype of protection and that of risk was the *C* allele of the rs3102735polymorphism. Considering that our data represent the first evidence in SA, additional studies are needed to validate the functional role of this genetic variant and its possible clinical impact in the progression of atherosclerosis in asymptomatic individuals. This study contributes to the knowledge in the field of molecular cardiology with the clinical interest of developing future panels of genetic markers of ischemic disease, in asymptomatic individuals, for use in preventing its progression.

## 5. Conclusions

Our data showed a lower genetic risk of SA in *C* allele carries of *OPG* (rs3102735) polymorphism. In our research, it was possible to distinguish one haplotype of risk and another haplotype of protection against SA. These results represent a first *OPG* genetic evidence in the field. However, additional studies that evaluate different populations are necessary to establish if these genetic markers are useful for a more accurate early screening of individuals at risk of asymptomatic atherosclerosis.

## Figures and Tables

**Table 1:** Characteristics of studied groups.

Metabolic and Clinical Characteristics *	Subclinical Atherosclerosis (*n* = 372)	Controls (*n* = 1041)	*p*
Age (years)	59 ± 8	51 ± 9	<0.0001
Gender (% male)	75.5	42.3	<0.0001
BMI (kg/m2)	28.2 [26.0–31.0]	27.8 [25.4–30.9]	<0.0001
Waist circumference	98 ± 11	94 ± 11	0.022
Triglycerides (mg/dL)	161 [120–221]	145 [107–202]	0.025
Glucose (mg/dL)	94 [86–105]	90 [84–97]	<0.0001
Alanine transaminase (IU/L)	24 [18–32]	24 [18–34]	0.391
Aspartate transaminase (IU/L)	25 [21–30]	25 [20–30]	0.596
Apolipoprotein AI (mg/dL)	133 [114–156]	134 [114–156]	0.804
Alkaline phosphatase (IU/L)	77 [64–92]	81 [68–96]	0.006
Serum calcium (mg/dL)	9.7 ± 0.5	9.7 ± 0.6	0.907
Serum phosphorus (mg/dL)	3.4 ± 0.5	3.5 ± 0.5	0.003
**Cardiovascular risk factors**			
Obesity (%)	32.8	30.5	0.434
Smoking habits (%)	21.5	22.9	0.613
Subcutaneous abdominal fat (%)	58.6	48.7	0.001
Hypoalphalipoproteinemia (%)	45.2	52.2	0.022
Non-HDL cholesterol > 160 mg/dL (%)	42.5	27.6	<0.0001
LDL-cholesterol ≥ 130 mg/dL (%)	44.9	28.8	<0.0001

Data are shown as mean ± standard deviation, median [interquartile range] or percentage. LDL = low-density lipoprotein, HDL = high-density lipoprotein. * Student’s *t*-test, Mann−Whitney U or Chi-squared.

**Table 2:** Description of analyzed sequence of polymorphisms studied.

SNPrsID	ID Assay	MAF	Analyzed Sequence
rs3102735	C___1971046_10	16%	CTCTAGGGTTCGCTGTCTCCCCCAT**[C/T]**AATTCCTGGTCTAGAAGTTAGACT
rs3134070	C__27466052_20	33%	TCCGCCCCAGCCCTGAAAGCGTTAA**[C/T]**CCTGGAGCTTTCTGCACACCCCCCG
rs3134069	C__27464534_20	10%	TTCCTACGCGCTGAACTTCTGGAGT**[A/C]**GCCTCCTCGAGGTCTTTCCACTAGC
rs2073618	C___1971047_40	33%	GGTTTCCGGGGACCACAATGAACAA**[C/G]**TTGCTGTGCTGCGCGCTCGTGGTAA

## Data Availability

The data described in this article can be requested from the corresponding author.

## References

[1] Posadas-Romero C., López-Bautista F. (2017). Prevalencia y extensión de la calcificación arterial coronaria en población mexicana asintomática cardiovascular: Estudio Genética de la Enfermedad Aterosclerosa. Arch. Cardiol. Mex..

[2] Acuña-Valerio J., Rodas-Díaz M.A. (2017). Aortic valve calcification prevalence and association with coronary risk factors and atherosclerosis in Mexican population. Arch. Cardiol. Mex..

[3] Faggiano A., Santangelo G. (2021). Cardiovascular Calcification as a Marker of Increased Cardiovascular Risk and a Surrogate for Subclinical Atherosclerosis: Role of Echocardiography. J. Clin. Med..

[4] Faggiano P., Dasseni N. (2019). Cardiac calcification as a marker of subclinical atherosclerosis and predictor of cardiovascular events: A review of the evidence. Eur. J. Prev. Cardiol..

[5] Li W., Su S.A. (2021). Emerging roles of fibroblasts in cardiovascular calcification. J. Cell. Mol. Med..

[6] Miller M. (2009). An emerging paradigm in atherosclerosis: Focus on subclinical disease. Postgrad. Med..

[7] Rochette L., Meloux A. (2019). The Role of Osteoprotegerin in Vascular Calcification and Bone Metabolism: The Basis for Developing New Therapeutics. Calcif. Tissue Int..

[8] Abdi S., Binbaz R.A. (2021). Association of RANKL and OPG Gene Polymorphism in Arab Women with and without Osteoporosis. Genes.

[9] Dutka M., Bobiński R. (2021). Osteoprotegerin and RANKL-RANK-OPG-TRAIL signalling axis in heart failure and other cardiovascular diseases. Heart Fail. Rev..

[10] Kiani A.N., Aukrust P. (2017). Serum osteoprotegrin (OPG) in subclinical atherosclerosis in systemic lupus erythematosus. Lupus.

[11] Dekker M., Waissi F. (2021). High levels of osteoprotegerin are associated with coronary artery calcification in patients suspected of a chronic coronary syndrome. Sci. Rep..

[12] Makarović S., Makarović Z. (2015). Osteoprotegerin and Vascular Calcification: Clinical and Prognostic Relevance. Coll. Antropol..

[13] Higgins C.L., Isbilir S. (2015). Distribution of alkaline phosphatase, osteopontin, RANK ligand and osteoprotegerin in calcified human carotid atheroma. Protein J..

[14] Luna-Luna M., Cruz-Robles D. (2017). Differential expression of osteopontin, and osteoprotegerin mRNA in epicardial adipose tissue between patients with severe coronary artery disease and aortic valvular stenosis: Association with HDL subclasses. Lipids Health Dis..

[15] Cottin Y., Issa R. (2021). Association between Serum Osteoprotegerin Levels and Severity of Coronary Artery Disease in Patients with Acute Myocardial Infarction. J. Clin. Med..

[16] Strobescu-Ciobanu C., Giuşcă S.E. (2020). Osteopontin and osteoprotegerin in atherosclerotic plaque—Are they significant markers of plaque vulnerability?. Rom. J. Morphol. Embryol..

[17] Pérez de Ciriza C., Moreno M. (2014). Circulating osteoprotegerin is increased in the metabolic syndrome and associates with subclinical atherosclerosis and coronary arterial calcification. Clin. Biochem..

[18] Hakimi M., Hyhlik-Dürr A. (2013). The expression of glycophorin A and osteoprotegerin is locally increased in carotid atherosclerotic lesions of symptomatic compared to asymptomatic patients. Int. J. Mol. Med..

[19] Miramontes-González J.P., Usategui-Martín R. (2019). VEGFR2 and OPG genes modify the risk of subclinical coronary atherosclerosis in patients with familial hypercholesterolemia. Atherosclerosis.

[20] Pleskovič A., Ramuš S.M. (2017). Polymorphism rs2073618 of the osteoprotegerin gene as a potential marker of subclinical carotid atherosclerosis in Caucasians with type 2 diabetes mellitus. Vasa.

[21] Villarreal-Molina T., Posadas-Romero C. (2012). The ABCA1 gene R230C variant is associated with decreased risk of premature coronary artery disease: The genetics of atherosclerotic disease (GEA) study. PLoS ONE.

[22] Posadas-Sánchez R., López-Uribe Á R. (2017). Association of the I148M/PNPLA3 (rs738409) polymorphism with premature coronary artery disease, fatty liver, and insulin resistance in type 2 diabetic patients and healthy controls. The GEA study. Immunobiology.

[23] Medina-Urrutia A., Posadas-Romero C. (2015). Role of adiponectin and free fatty acids on the association between abdominal visceral fat and insulin resistance. Cardiovasc. Diabetol..

[24] Hernández-Avila M., Romieu I. (1998). Validity and reproducibility of a food frequency questionnaire to assess dietary intake of women living in Mexico City. Salud Publica Mex..

[25] Mautner G.C., Mautner S.L. (1994). Coronary artery calcification: Assessment with electron beam CT and histomorphometric correlation. Radiology.

[26] Messeguer X., Escudero R. (2002). PROMO: Detection of known transcription regulatory elements using species-tailored searches. Bioinformatics.

[27] Xu Z., Taylor J.A. (2009). SNPinfo: Integrating GWAS and candidate gene information into functional SNP selection for genetic association studies. Nucleic Acids Res..

[28] Zhao H., Cao Y. (2017). The association between OPG rs3102735 gene polymorphism, microembolic signal and stroke severity in acute ischemic stroke patients. Gene.

[29] Soufi M., Schoppet M. (2004). Osteoprotegerin gene polymorphisms in men with coronary artery disease. J. Clin. Endocrinol. Metab..

[30] Pérez-Hernández N., Posadas-Sánchez R. (2020). Genetic Variants and Haplotypes in OPG Gene Are Associated with Premature Coronary Artery Disease and Traditional Cardiovascular Risk Factors in Mexican Population: The GEA Study. DNA Cell Biol..

[31] Rhee E.J., Oh K.W. (2006). The relationship between four single nucleotide polymorphisms in the promoter region of the osteoprotegerin gene and aortic calcification or coronary artery disease in Koreans. Clin. Endocrinol..

[32] Alkady E.A., Selim Z.I. (2020). Association of serum osteoprotegerin and osteoprotegerin gene polymorphism with subclinical carotid artery atherosclerosis and disease activity in rheumatoid arthritis patients. Egypt. Rheumatol..

[33] Barquera R., Hernández-Zaragoza D.I. (2020). The immunogenetic diversity of the HLA system in Mexico correlates with underlying population genetic structure. Hum. Immunol.

[34] Del Angel-Pablo A.D., Juárez-Martín A.I. (2020). HLA Allele and Haplotype Frequencies in Three Urban Mexican Populations: Genetic Diversity for the Approach of Genomic Medicine. Diagnostics.

[35] Salazar-Flores J., Dondiego-Aldape R. (2010). Population structure and paternal admixture landscape on present-day Mexican-Mestizos revealed by Y-STR haplotypes. Am. J. Hum. Biol..

[36] Juárez-Cedillo T., Zuñiga J. (2008). Genetic admixture and diversity estimations in the Mexican Mestizo population from Mexico City using 15 STR polymorphic markers. Forensic Sci. Int. Genet..

[37] Luna-Luna M., Zentella-Dehesa A. (2020). Epicardial Adipose Tissue in the Progression and Calcification of the Coronary Artery Disease. Biochemistry of Cardiovascular Dysfunction in Obesity.

[38] Min H., Morony S. (2000). Osteoprotegerin reverses osteoporosis by inhibiting endosteal osteoclasts and prevents vascular calcification by blocking a process resembling osteoclastogenesis. J. Exp. Med..

[39] Schoppet M., Preissner K.T. (2002). RANK ligand and osteoprotegerin: Paracrine regulators of bone metabolism and vascular function. Arterioscler. Thromb. Vasc. Biol..

[40] Gunes M., Temizkan S. (2021). Serum osteoprotegerin levels, endothelial function and carotid intima-media thickness in type 2 diabetic patients. J. Diabetes Complicat..

[41] Krzanowski M., Krzanowska K. (2018). Elevated Circulating Osteoprotegerin Levels in the Plasma of Hemodialyzed Patients With Severe Artery Calcification. Ther. Apher. Dial..

[42] Gaudio A., Privitera F. (2014). Relationships between osteoprotegerin, receptor activator of the nuclear factor kB ligand serum levels and carotid intima-media thickness in patients with type 2 diabetes mellitus. Panminerva Med..

[43] Akinci B., Demir T. (2008). Serum osteoprotegerin is associated with carotid intima media thickness in women with previous gestational diabetes. Diabetes Res. Clin. Pract..

[44] Kalvakolanu D.V., Roy S.K. (2005). CCAAT/enhancer binding proteins and interferon signaling pathways. J. Interferon Cytokine Res..

